# Resting State Functional Connectivity is Associated With Treatment Response in Major Depression: A Real World Study

**DOI:** 10.1192/j.eurpsy.2023.1266

**Published:** 2023-07-19

**Authors:** Y. Harrington, M. Paolini, V. Bettonagli, F. Colombo, S. Poletti, R. Zanardi, F. Benedetti

**Affiliations:** 1 IRCCS San Raffaele Scientific Institute; 2 Vita Salute San Raffaele University, Milan, Italy

## Abstract

**Introduction:**

Major depressive disorder (MDD) is largely considered the most prevalent psychiatric disorder worldwide. Despite its domineering presence, effective treatment for many individuals remains elusive. Investigation into relevant biological markers, specifically neuroimaging correlates, of MDD and treatment response have gained traction in recent years; however, findings are still inconsistent.

**Objectives:**

In this study, we aimed to investigate the resting state functional connectivity patterns associated with treatment response in MDD inpatients in a real world setting.

**Methods:**

Forty-three inpatients suffering from a major depressive episode were recruited from the psychiatric ward at IRCCS San Raffaele Hospital in Milan, Italy. Symptom severity was assessed via the 21-item Hamilton Depression Rating Scale (HDRS). The percentage of decrease in HDRS scores from admission to discharge was then calculated with the formula [(HDRS admission – HDRS discharge) * 100] / HDRS admission. All patients underwent a 3T MRI scan within one week of admission to acquire resting-state fMRI images, which included 200 sequential T2*-weighted volumes. Images were preprocessed using the CONN toolbox, running within Statistical Parametric Mapping (SPM 12). Preprocessing was performed according to a standard pipeline. A voxel-wise metric, intrinsic connectivity contrast (ICC), was implemented to explore the global resting state functional connectivity (rs-FC) patterns associated with treatment response. ICC-derived maps were then entered in the second-level analyses to examine the effect of the percentage of HDRS decrease, including age, sex, admission HDRS score, duration of hospitalization, and antidepressant dose equivalents as nuisance covariates.

**Results:**

We found that the percentage of HDRS decrease after treatment predicted rs-FC. ICC analysis identified 2 clusters where changes in HDRS scores were significantly associated with rs-FC, with increased connectivity in the supramarginal gyrus (pFDR = 0.002) and decreased connectivity in the amygdala and parahippocampal gyrus (pFDR = 0.047).

**Conclusions:**

Our results suggest that altered connectivity of the supramarginal gyrus, amygdala and parahippocampal gyrus is related to antidepressant treatment response. Given that these brain areas are implicated in emotional processing and mood, it is conceivable that a better integrity of brain connectivity may facilitate treatment response in major depression.

**Disclosure of Interest:**

None Declared

